# Celebrating a year of change and progress

**DOI:** 10.3390/curroncol13060020

**Published:** 2006-12

**Authors:** M. McLean

As 2006 draws to a close, *Current Oncology* is marking its most successful year to date, with a record number of manuscripts submitted and a record number published. We are close to maintaining our objective of an eight-week turnaround for acceptances, and our electronic submission software at www.current-oncology.com is proving robust—although not without the occasional “issue.” But we are advised that an improved version is in the offing.

The Editorial Board continues to grow in both quantity and quality, and I am particularly pleased to welcome Dr. Phil Gold, Douglas G. Cameron Professor of Medicine, McGill University, as Deputy Editor.

Ongoing support from industry in the form of advertising revenue and unrestricted educational grants is a primary reason that *Current Oncology* continues to grow in strength and can be delivered without charge to its readership. The publisher and I therefore take this opportunity to express our gratitude for this support.

From everyone at *Current Oncology,* best wishes to all for a happy and prosperous holiday season.

